# Operando Laboratory X-Ray Imaging of Silver-Based Gas Diffusion Electrodes during Oxygen Reduction Reaction in Highly Alkaline Media

**DOI:** 10.3390/ma12172686

**Published:** 2019-08-22

**Authors:** Melanie Cornelia Paulisch, Marcus Gebhard, David Franzen, André Hilger, Markus Osenberg, Nikolay Kardjilov, Barbara Ellendorff, Thomas Turek, Christina Roth, Ingo Manke

**Affiliations:** 1 Institute of Applied Materials, Helmholtz-Zentrum Berlin, Hahn-Meitner-Platz 1, 14109 Berlin, Germany; 2 Institute of Chemistry and Biochemistry, Freie Universität Berlin, Takustraße 3, 14195 Berlin, Germany; 3 Institute of Chemical and Electrochemical Process Engineering, Clausthal University of Technology, Leibnizstrasse 17, 38678 Clausthal-Zellerfeld, Germany; 4 Department of Materials Science and Technology, Technical University Berlin, Hardenbergstr. 36, 14109 Berlin, Germany

**Keywords:** operando, X-ray imaging, gas diffusion electrode, oxygen-depolarized cathode, oxygen reduction reaction, chlor-alkali electrolysis

## Abstract

Operando laboratory X-ray radiographies were carried out for imaging of two different silver-based gas diffusion electrodes containing an electroconductive Ni mesh structure, one gas diffusion electrode composed of 95 wt.% Ag and 5 wt.% polytetrafluoroethylene and one composed of 97 wt.% Ag and 3 wt.% polytetrafluoroethylene, under different operating parameters. Thereby, correlations of their electrochemical behavior and the transport of the 30 wt.% NaOH electrolyte through the gas diffusion electrodes were revealed. The work was divided into two parts. In the first step, the microstructure of the gas diffusion electrodes was analyzed ex situ by a combination of focused ion beam technology and synchrotron as well as laboratory X-ray tomography and radiography. In the second step, operando laboratory X-ray radiographies were performed during chronoamperometric measurements at different potentials. The combination of the ex situ microstructural analyses and the operando measurements reveals the impact of the microstructure on the electrolyte transport through the gas diffusion electrodes. Hence, an impact of the Ni mesh structure within the gas diffusion electrode on the droplet formation could be shown. Moreover, it could be observed that increasing overpotentials cause increasing electrolyte transport velocities and faster droplet formation due to electrowetting. In general, higher electrolyte transport velocities were found for the gas diffusion electrode with 97 wt.% Ag in contrast to that with 95 wt.% Ag.

## 1. Introduction

Chlorine is an extensively used and widely needed chemical product. However, the production is based on the chlor-alkali electrolysis, which is responsible for high energy demand [1]. During the state-of-the-art membrane process chlorine gas is produced at the anode, while, at the cathode in conjunction with hydroxide ion production, hydrogen gas evolves. In order to reduce the electrical energy demand, and thus the corresponding CO_2_ emissions, significant success was achieved by using metallic gas diffusion electrodes (GDE) [2], which catalyze oxygen reduction instead of hydrogen evolution, lowering the necessary cell voltage by approximately one volt. This process is called oxygen depolarized cathode (ODC) technology and may lead to electrical energy savings of up to 30% [3], which will help to comply with future emission limits [1,4,5]. With respect to the application in highly alkaline electrolytes, the required high activity as well as cost efficiency, porous silver-based GDEs were developed [6,7]. The GDEs are composed of silver grains, a complex surrounding pore system and polytetrafluoroethylene (PTFE) at the silver grain boundaries [8]. While the hydrophilic silver grains are supporting the penetration of the 30 wt.% NaOH electrolyte, pore channels, which contain a high hydrophobic PTFE amount, are expected to be kept free for the gas phase [7]. Additionally, the GDEs contain a nickel mesh for enhancing the electrical conductivity. Franzen et al. [8] investigated silver-based gas diffusion electrodes with different Ag to PTFE ratios, starting with 90 wt.% Ag and 10 wt.% PTFE up to 99 wt.% Ag and 1 wt.% PTFE, regarding the changes of their pore system and their electrochemical performance. It was shown that the formation of the silver grain skeleton is hardly influenced by the different compositions. However, with increasing PTFE content, more PTFE is deposited at the grain boundaries and thus the pore paths are narrowed. For electrodes with 90 wt.% Ag and 10 wt.% PTFE, it is almost closed. In correlation to the microstructural changes, the authors observed an increasing of the electrochemical performance with decreasing PTFE content, finding a maximum at 98 wt.% Ag and 2 wt.% PTFE. Knowledge about electrolyte distribution and transport in electrode materials is a key issue for their deeper understanding. The liquids, the free space available for the gas flow and the electrode material itself are forming the so-called three-phase boundary. Only at (and due to diffusion around) this boundary all three components are necessary for the chemical reactions present at the same time. However, until now, it is not completely understood how the electrolyte and the gas phase are distributed within the GDEs and how exactly the three-phase boundary form. In this context, it is not clear whether the three-phase boundary only forms in the first few micrometers of the GDE’s surface, which is in direct contact to the electrolyte or if there are percolating pore paths that provide a transport of the electrolyte through the electrode. Therefore, it is primarily important to investigate and understand how the microstructure impacts the distribution and transport of the electrolyte and the gas phase and hence the electrochemical properties of the GDEs. In the past, there have been extensive in situ and operando studies about the water distribution in polymer electrolyte membrane fuel cells [9,10,11,12,13,14,15]. However, much less is known about the electrolyte distribution in silver-based GDEs. To get more information about the impact of the microstructure on the electrochemical properties, an operando cell setup was designed and presented by Gebhard et al. [16]. With this cell design and sample environment, it was possible to get first results of the electrolyte transport within the GDEs recorded by laboratory X-ray imaging in realistic operation conditions. This work is directly based on the studies of Gebhard et al. [16] and presents further analysis regarding how the microstructure impacts the electrolyte transport and the droplet formation. Furthermore, the chronoamperometric tests were performed at different potentials to analyze the impact of the overpotentials on the electrochemical behavior and the electrolyte transport through the GDE.

## 2. Materials and Methods

### 2.1. Preparation of Silver-Based Gas Diffusion Electrodes

The GDEs that were analyzed in this work were produced at Clausthal University of Technology at the Institute of Chemical and Electrochemical Process Engineering. For the GDEs, a nickel mesh was utilized, which supports the electrical conductivity. The Ni mesh (Haver and Boecker OHG, Oelde, Germany) has a mesh size of 106 µm × 118 µm and a wire thickness of 63 µm. Furthermore, a suspension with silver particles (SF9ED, Ferro GmbH, Frankfurt a. M., Germany) and a PTFE dispersion (PTFE Dispersion TF 5060GZ, 3M™ Dyneon™, Burgkirchen, Germany) was mixed. As thickener and pore building agent, a methyl cellulose solution with 1 wt.% hydroxyethylmethyl cellulose (WALOCEL™ MKX 70000 PP 01, Bomlitz, Germany) and deionized water was admixed. Eighty layers of the mixture were sprayed on the Ni mesh using a spray piston with a pin hole of 0.6 mm (Evolution, Harder & Steenbeck, Norderstedt, Germany). By spraying 80 layers, a reproducible production of the electrodes is ensured; in general, a catalyst loading of 150 mg·cm^−2^ is obtained. To ensure a homogeneous surface, a simultaneous drying process is indispensable. Therefore, the spraying process was performed on a heating table [17]. After every sprayed layer, the GDE has to dry completely before the next layer can be applied, in order to assure a homogeneous surface. To burn out the methyl cellulose and to increase the mechanical stability, the sprayed GDEs were hot pressed at 15 MPa and 130 °C for 5 min and heat treated for 15 min at 330 °C. Finally, the GDEs have a chemical composition of 97 wt.% Ag + 3 wt.% PTFE or 95 wt.% Ag + 5 wt.% PTFE. The thicknesses of the GDEs were measured at six different areas with a dial gauge (FD 50, Käfer GmbH, Villingen-Schwenningen, Germany). The GDE with 95 wt.% Ag exhibits a thickness of 294 ± 16 µm and the GDE with 97 wt.% Ag exhibit a thickness of 300 ± 19 µm. The GDE with 97 wt.% Ag was chosen for this investigation because it is thoroughly investigated and offers a stable performance [7,8,17]. The GDE with 95 wt.% Ag was chosen because it is known to have a weaker performance [8]. Analyzing both GDEs, the electrolyte’s distribution behavior during operando measurements is assessed. To obtain 3D structure information of the pristine GDEs, ex situ synchrotron tomography was applied. However, due to the high X-ray absorption by nickel, it is not possible to measure the GDE with the nickel mesh. For this purpose, a GDE without Ni mesh was produced especially for synchrotron tomography, in order to reduce the absorption and enable the radial radiolucency. In this case, a mixture of 99 wt.% Ag and 1 wt.% PTFE was sprayed on a Kapton^®^ film that was removed after the whole preparation process. The sintering and heat treatment processes were the same as described before. The thickness of this GDE was about 120 µm. The microstructure of this specimen is expected to be similar to that of the GDEs containing the Ni mesh, with the exception of the layers that are surrounding the Ni mesh directly. Franzen et al. [8] show that the ratio of Ag to PTFE has less influence on the silver skeleton. Moreover, the PTFE absorption is too low to show any contrast in the synchrotron tomography. Therefore, it is possible to use this specimen to get reliable results about the 3D microstructure. The specimen was cut by scalpel to a length of 10 mm and grinded down to a small square rod with a cross section of 120 µm × 120 µm.

### 2.2. Electrochemical Measurement

**Figure 1:**
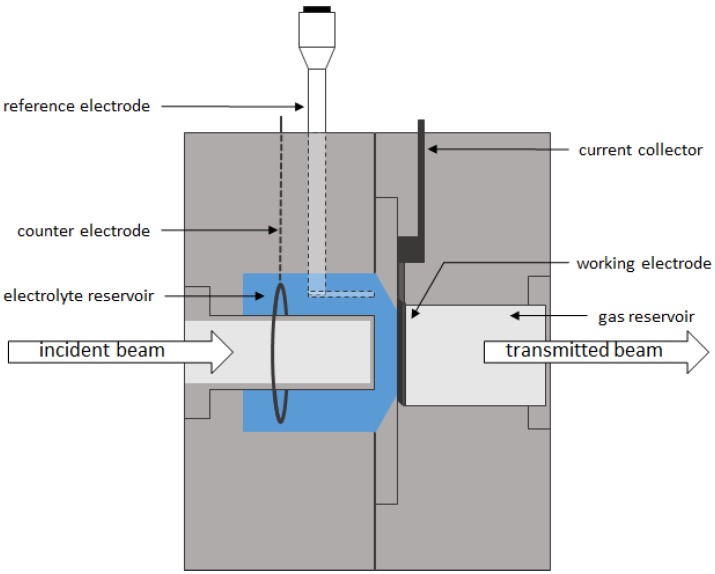
Schematic illustration of the half-cell setup. It is shown in more detail and described by Gebhard et al. [16].

To analyze the behavior of the 30 wt.% NaOH electrolyte flow within the GDE during electrochemical operation, a half-cell compartment was constructed by Freie Universität Berlin. The half-cell compartment is specially designed for simultaneous electrochemical characterization and operando X-ray radiography. Figure 1 shows the half-cell setup. The detailed construction is described by Gebhard et al. [16].

The operando measurements were carried out at room temperature using an O_2_ gas flow of 20 mL/min and 250 mL of 30 wt.% NaOH electrolyte with a flow of 1 L/h. The electrolyte was circulated several times in a closed circuit. Electrochemical potentials were measured versus a reversible hydrogen electrode (RHE) in a three-electrode setup. The GDE that is to be analyzed is the working electrode. Here, oxygen and water together with electrons are reduced to hydroxide ions:

O_2_ + 2H_2_O + 4e^−^ → 4OH^−^  E^ø^ = 0.40 V (vs. NHE)

The GDEs were tested according to the following chronoamperometric procedure: for 60 min at 0.9 V vs. RHE, 0.5 V vs. RHE and 0.2 V vs. RHE, respectively. The chronoamperometric tests were performed four times. The field of view and the geometrical surface area had a size of 3.14 cm^2^. The results are not corrected regarding the internal resistance of the cell.

### 2.3. Focused Ion Beam

To analyze the pore system in more detail, a high spatial resolution of the microstructure is required. Therefore, scanning electron microscopy (SEM) and focused ion beam (FIB) technology were applied to pristine GDEs, using a Zeiss Crossbeam 340 Gallium-FIB/SEM (Oberkochen, Germany). To analyze the cross section, a sample piece of 3 mm × 3 mm was cut from the center of the GDE and vertically mounted between two parallel plates perpendicularly orientated to the ion beam. The cross-sectional surface was ion milled. Furthermore, a piece of 3 mm × 3 mm was taken mechanically from the center of the GDE and horizontally fixed on a sample holder using a carbon pad. For the coarse cut, 30 keV and 30 nA, and, for the polishing step, 30 keV and 700 pA were applied.

### 2.4. Laboratory X-Ray Measurements

The laboratory X-ray tomography and the operando radiography measurements were carried out with a Hamamatsu X-ray source (tungsten anode, Hamamatsu City, Japan) and a Hamamatsu detector (Typ C7942SK-05, Hamamatsu City, Japan) (part of the CONRAD µ-CT Laboratories at Helmholtz-Zentrum Berlin [18,19]). The tomography of a pristine GDE was done at 150 kV and 66 µA with a 0.5 mm thick Cu filter employing an exposure time of 2.6 s using 3 frames for each projection for noise reduction. The operando processes were investigated with 130 kV and 230 µA. The images were exposed for 2.5 s in case of 95 wt.% Ag, for 2.8 s in case of 97 wt.% Ag, with 3 frames and a pixel size of 12 µm. The radiographic projections from the operando tests were normalized to the reference state of the cell, filled with electrolyte but without applied potential. Hence, differences of the liquid transport in the GDE become visible and can be quantified as liquid column applying the Lambert–Beer law. To analyze the evolution of droplets for defined times, subtraction images were calculated. Therefore, the events where a previously formed droplet was dropping down, from now on called “drop down”, was determined and two stacks, 100 images before and 100 images after it, were each averaged in the time domain. Subsequently, the averaged image, taken before the drop down, was subtracted from the averaged image, taken after it. Hence, differences that occur in the chosen period of time become visible. The calculations were performed with the imaging software Fiji (ImageJ 1.52p) [20,21].

### 2.5. Synchrotron

For analyzing the ex situ 3D microstructure in a larger volume, pristine GDEs were analyzed by synchrotron X-ray imaging. The GDEs that contain the Ni mesh were measured by radiography (2D) and the specimen that was sprayed on Kapton^®^ film was analyzed by tomography (3D). The measurements were carried out at the BAMline at BESSY II. To keep the X-ray absorption as low as possible and due to the energy limit of the beamline at 40 keV, a monochromatic photon energy of 25 keV, tightly beneath the K edge, was applied. The optical setup consisted of a (4008 × 2672) pixel CCD camera (PCO 4000, PCO AG, Kehlheim, Germany) with a CdWO_4_ scintillator screen in combination with an Optique Peter lens setup (Optique Peter, Lentilly, France) providing a pixel size of 438 nm. 3D tomography was used for further analyses of the pore distribution within the electrodes. The corresponding calculations were also done with the imaging software Fiji (ImageJ 1.52p) [20,21].

## 3. Results

### 3.1. Ex Situ Analysis of the Microstructure

**Figure 2:**
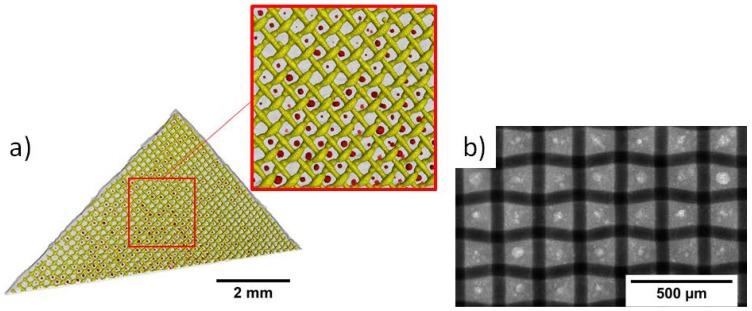
Microstructure of the Ag-based GDE, Ni mesh and pores within the Ag: (**a**) laboratory X-ray tomography; Ni mesh is indicated yellow, the silver in grey and pores in red (**b**) synchrotron radiography.

To analyze the microstructure, pristine GDEs were characterized by laboratory X-ray tomography and synchrotron radiography (Figure 2). Figure 2a shows the regular pattern of the Ni mesh (yellow) placed in the Ag electrode (grey). Within the interspaces of the Ni mesh, red globular areas of about 40 µm are visible, which indicate larger pores in the GDE. The synchrotron radiography (Figure 2b) shows the microstructure in more detail. Here, the Ni mesh is dark grey. On it and in its interspaces, Ag becomes visible in a brighter grey than the mesh. Again, larger pores within the Ag are visible in the interspaces of the Ni mesh.

**Figure 3:**
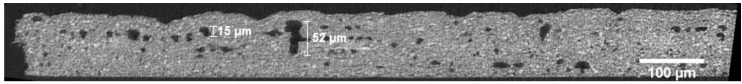
Synchrotron tomography, microstructure of Ag-based GDE sprayed on Kapton^®^ film (bottom side), Ag with pores.

To characterize the 3D microstructure, the GDE sprayed on Kapton^®^ film was analyzed by synchrotron tomography. Figure 3 shows a reconstructed 2D slice, which shows a heterogeneous distribution of irregular pores. The largest pore is 52 µm in length. However, it is also visible that larger pores form occasionally.

**Figure 4:**
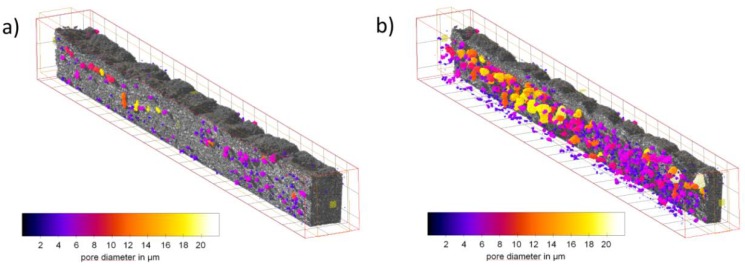
Synchrotron tomography, microstructure of Ag-based GDE sprayed on Kapton^®^ film, Ag (grey) with pores, which are colored in accordance with their size (see legend); (**a**) pores within GDE; (**b**) silver GDE subtracted to give visibility to the pores.

Figure 4 shows the 3D reconstructed data set of the tomography. The Ag phase is indicated in grey while the pores are colored corresponding to their lookup table (Figure 4). The PTFE distribution is not visible due to the low absorption of PTFE in comparison to Ag and Ni.

**Figure 5:**
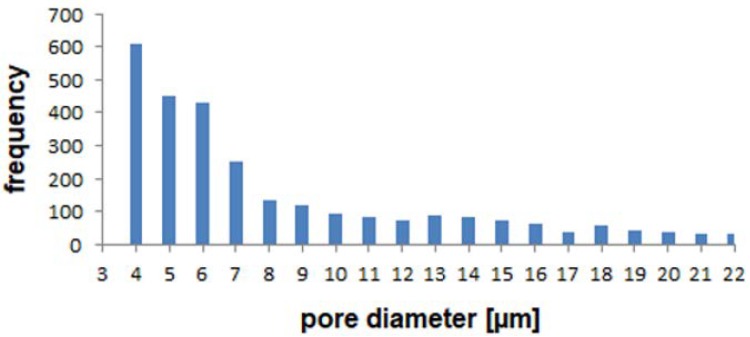
Pore size distribution in the Ag-based GDE sprayed on Kapton^®^ film.

For more detailed analyses of the pore system, the pores were fitted with spheres. The calculated pore diameter distribution obtained with this procedure is shown in the histogram (Figure 5). Due to deviations in the optical system of the synchrotron and the data filtering and processing, it has to be noted that the smallest clearly measurable value taken into account is 4 µm. Therefore, values lower than 4 µm were not taken into account and excluded from the histogram. 

The histogram shows that the main fraction of the measurable pores has a size of 4 µm. Furthermore, pores of 5 µm and 6 µm size show a considerable fraction. Larger pores show lower frequencies. Due to the fact that the maximum fraction of the pores is 4 µm, which indicates the smallest value that can be measured clearly, smaller pores than 4 µm are to be expected. Therefore, a more detailed look into the microstructure is necessary, which can be realized by using FIB/SEM.

**Figure 6:**
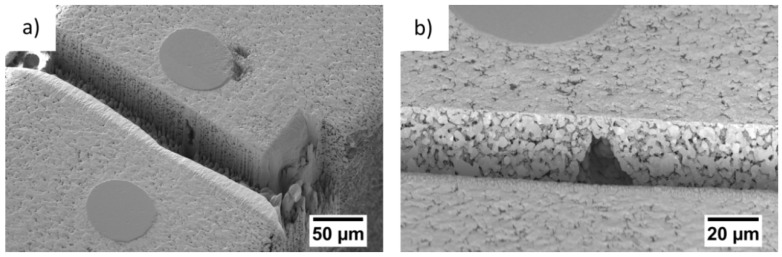
FIB/SEM, microstructure of Ag-based GDE: (**a**) cross-sectional cut through whole GDE; (**b**) higher magnification of large pore between Ni wires.

First, a FIB cut over the whole cross section of the GDE was made (Figure 6). The sample surface shows the cross section of two Ni wires while the cut was placed between them. Close to the wires, a large pore is visible (Figure 6a). Figure 6b shows the large pore in higher magnification. Although there is a lot of Ag redeposition within the pore, which deposits during the FIB cutting, it is also visible that the pore is connected to the smaller pore system in the surrounding microstructure. Apart from that, the cross-sectional cut shows a homogeneous microstructure.

**Figure 7:**
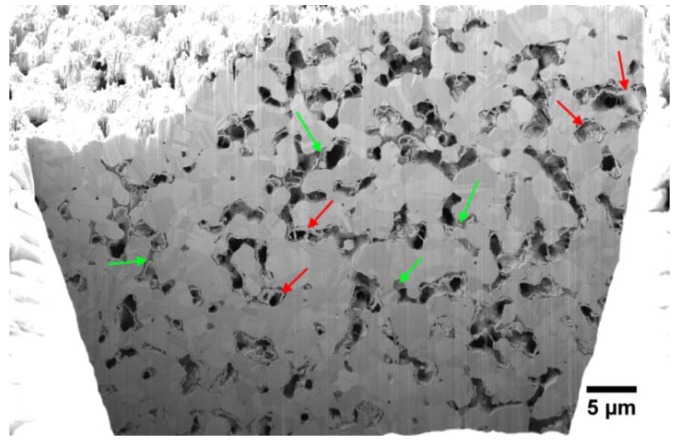
FIB/SEM, microstructure of Ag-based GDE, Ag grains with twins, dark grey PTFE deposition (green arrows) and Ag redeposition (red arrows). Within the material, a complex pore system is visible.

Figure 7 shows the microstructure of the GDE in more detail. Within the grey silver grains, twinned crystallites have formed. The PTFE (dark grey, green arrows) is deposited on the silver grain boundaries. In addition, Ag redeposition is visible as light grey lines (red arrows) at the PTFE and the Ag grains. The FIB cut also shows that the pores are not closed but form a complex pore system.

### 3.2. Operando Radiography of the Electrochemical Process

**Figure 8:**
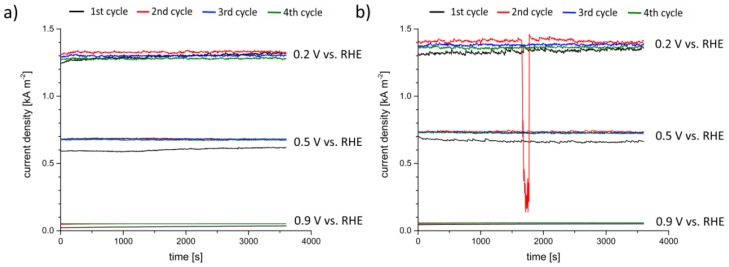
Chronoamperometry curves: (**a**) GDE with 95 wt.% Ag; (**b**) GDE with 97 wt.%.

For the operando measurements, two GDEs with 95 wt.% and 97 wt.% Ag were used. Figure 8 shows the results of chronoamperometric measurements for both GDEs.

The chronoamperometry curves of the GDE with 95 wt.% Ag are shown in Figure 8a. The measurements at 0.9 V vs. RHE show for each cycle steady current density curves over the whole recording time. Moreover, it is visible that the first cycle indicates lower current densities than the others; however, the current densities of the last three cycles are almost identical. The current densities obtained at 0.5 V vs. RHE exhibit the same characteristics. Those taken at 0.2 V vs. RHE reveal higher differences between them than those observed at lower overpotentials. It is obvious that the current densities of the first and the last cycle are the lowest for this potential. The best results were observed in the second cycle. Moreover, the current densities obtained at 0.2 V vs. RHE exhibit higher irregularities than those at 0.5 V vs. RHE and 0.9 V vs. RHE.

The chronoamperometry curves of the GDE with 97 wt.% Ag (Figure 8b) are similar to those of the GDE with 95 wt.% Ag. The current densities taken at 0.9 V vs. RHE are almost the same for each cycle and steady over the whole measuring time. The current densities obtained at 0.5 V vs. RHE are similar to each other; however, the current densities recorded for the first cycle are lower than for the other cycles. At 0.2 V vs. RHE, the second cycle shows the highest current density; however, a strong current density dip is visible between 1600 s and 1750 s.

**Figure 9:**
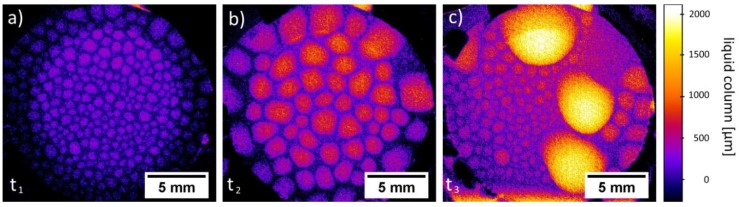
Laboratory X-ray, operando measurement of GDE with 97 wt.% Ag with half-cell compartment: (**a**) t_1_ = 0 s, evenly spread droplets at the electrode’s gas side; (**b**) t_2_ = t_1_ + 02 h 07 min 23 s, grown and coagulated droplets; (**c**) t_3_ = t_1_ + 06 h 45 min 21 s, new droplets form evenly spread in newly free areas.

Regarding the laboratory X-ray measurements, for both GDEs, the same behavior during the experiments was observed: after approximately two hours, the first droplets form evenly spread over the electrode’s gas side (Figure 9a). In a further process, the droplets grow (Figure 9b), coagulate (Figure 9c, right side) and fall down. In those areas freed from covering droplets, new droplets form evenly spread over this area. (Figure 9c, left side) and the droplet formation cycle starts again. During the operando measurement, both GDEs passed this cycle twice.

**Figure 10:**
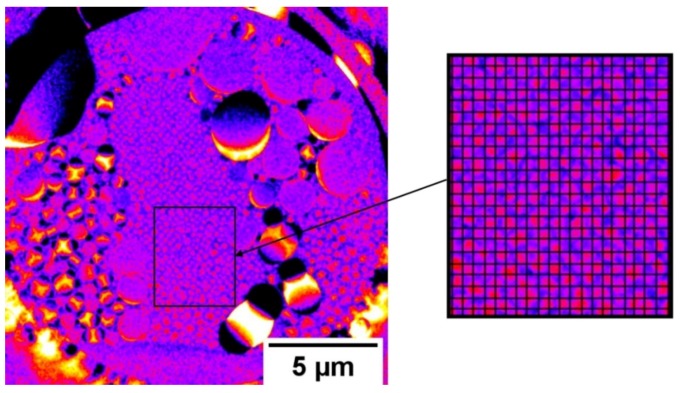
Laboratory X-ray, operando measurement of GDE with 95 wt.% Ag with half-cell compartment, subtraction image, overview about liquid changes after first large droplets dropped down and higher magnification with calculated and added Ni mesh, showing newly formed droplets in the area, where the first coarse droplets dropped down.

To analyze the new forming droplets, subtraction images were calculated after the first large droplets fell down (Figure 10). This allows for imaging the changes of the liquid distribution after the drop down until new small droplets form. This gives us direct access to the speed of liquid transport through the GDE. Furthermore, the place of the Ni mesh within the electrode was calculated and added to the subtraction picture (higher magnification on the right side in Figure 10). Therefore, an algorithm was written, which filtered the mesh position from the original images and created a binary image of the mesh. Finally, superpositions of the binary mesh images and the normalized images were calculated.

For small new formed droplets, which have the same size as the mesh size, Figure 9b shows that they have the same spatial periodicity as the mesh. Droplets, which are already grown and are larger than the mesh size, are also positioned on the mesh wires. Moreover, within some mesh interspaces, no drops are visible.

**Figure 11:**
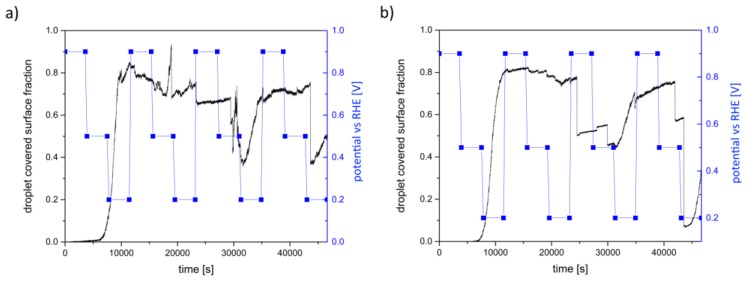
Droplet covered surface fraction on the gas side of the GDE and applied potentials: (**a**) GDE with 95 wt.% Ag; (**b**) GDE with 97 wt.% Ag.

To investigate the relationship between the droplet formation and the applied potential, the droplet covered area on the gas side of the GDE was measured. Figure 11 shows the area fraction that is covered with droplets and how the covering is affected by the applied potentials for both GDEs versus time. This analysis also gives information about the droplet free surface area, which is available for gas penetration. For the calculation, the whole field of view was used.

For the GDE with 95 wt.% Ag, it is obvious that, during periods while 0.9 V vs. RHE is applied, the area fraction is decreasing or steady. This means that, while this potential is applied in the first cycle, no droplets form. In the second cycle, the area fraction is reduced due to the agglomeration of several smaller droplets to less larger ones, which is reducing the occupied surface. During the third and fourth cycle, the droplet covered surface area is almost the same. For the periods when 0.2 V vs. RHE is applied, the covered surface area is increasing significantly. This is especially visible for the first cycle, when the droplets form for the first time and for the third and fourth cycle, when the droplets form new after a drop has fallen down.

The GDE with 97 wt.% Ag (Figure 11b) shows the same behavior: While the area fraction of the droplets covered surface is almost constant at 0.9 V vs. RHE, a significant increase is visible for 0.2 V vs. RHE. However, the drop down of the droplets is not synchronized with the change of the potential. 

**Figure 12:**
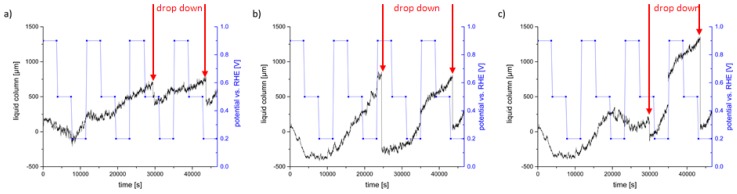
Saturation curves of the GDEs and applied potentials; red arrows indicate drop down: (**a**) GDE with 95 wt.% Ag; (**b**) GDE with 97 wt.% Ag, ROI 1; (**c**) GDE with 97 wt.% Ag, ROI 2.

In addition to the information about the liquid distribution on the gas side surface of the electrode (Figure 11), the liquid transportation through the thickness of the electrode and the droplets was analyzed by calculating saturation curves for both GDEs. In this case, the saturation is defined as liquid transport through the GDE and the droplets referred to the measured time. The images are normalized to the reference state of the cell, filled with electrolyte but without applied potential. In all investigated cases, the drop down does not concern the whole surface of the GDE; therefore, for both GDEs, different regions of interest (ROI) with the same area size were analyzed, where the drop downs are significantly visible. The *y*-axes of these diagrams are set as a liquid column and show how much liquid is located within the electrode and in droplets in the chosen ROIs. This analysis gives information about how much liquid can enter and pass the electrode and how this is affected by the applied potentials. While the GDE with 95 wt.% Ag shows similar behaviors in the different ROIs, considerable differences are observed for the GDE with 97 wt.% Ag. Figure 12 shows the saturation characteristics for both GDEs. In case of the GDE with 95 wt.% Ag, one representative curve is shown (Figure 12a), in case of the GDE with 97 wt.% Ag, the saturation curves of two different ROIs are presented (Figure 12b,c).

Figure 12 shows the saturation curve of the GDE with 95 wt.% Ag for the whole electrochemical process with the significant aspects being listed in Table 1.

Figure 12b,c show the saturation curves of the GDE with 97 wt.% Ag during the whole electrochemical process for two different ROIs. Below, the significant aspects are listed for the first ROI (Figure 12b) in Table 2.

The saturation behaviour in the second ROI (Figure 12c) is similar to those described for the first. However, at 0.2 V vs. RHE in the second cycle and 0.9 V vs. RHE of the third cycle, a sudden decrease of the saturation is visible. After the first coarse droplets dropped down at 0.5 V vs. RHE in the third cycle, the saturation behaviour is similar to that described for the first ROI. The meaning of this behaviour will be discussed in the following.

## 4. Discussion

### 4.1. Microstructure of the Gas Diffusion Electrodes

The microstructure of the GDEs shows silver grains with deposited PTFE at their grain boundaries and a complex open pore system extending through the whole electrode (Figure 7). Previous investigations showed that this microstructure is homogenous over the whole cross section of the GDE [22]. Moreover, the laboratory X-ray radiographies and the FIB cuts demonstrate that large pores with sizes of about 40 µm form regularly between the Ni mesh (Figure 2). These pores are not closed but connected with the rest of the pore system. 

### 4.2. Electrolyte Distribution during Electrochemical Tests

It is known from the literature [7,8] that silver-based GDEs contain hydrophilic silver grains and hydrophobic PTFE deposits. The silver grains benefit the penetration of electrolyte, while the PTFE deposits act as obstacles for the electrolyte. Therefore, PTFE rich pore paths are kept free for the gas phase. Moreover, in this investigation, it was shown that, during the electrochemical tests, droplet formation can be observed. As visible in Figure 10, small droplets form on the gas side. This formation is mainly following the same spatial periodicity as the Ni mesh. However, it is to be noted that some silver areas between the mesh wires do not show large pores. Furthermore, the small droplets, which are formed on the surface of the GDE which is exposed to the gas side, do not cover some of these areas. The compliant behavior is to be observed for small droplets with diameters of some micrometers. As soon as the droplets grow and coagulate, they are losing the periodicity of the Ni mesh. In agreement with similar measurements of Haußmann et al. [23] and Alink et al. [24], it is to expect that the main electrolyte flow is realized by larger pore channels due to their reduced flow resistance. Therefore, we also assume that the large pores allow or at least positively affect the percolation of the electrolyte through the GDE and support the formation of droplets. Moreover, we expect that the small droplets are formed due to the liquid transport through percolating pore paths. However, the measurements in the laboratory X-ray show a high signal-to-noise ratio. To get more information about the droplet formation with higher spatial resolution, investigations in a synchrotron facility are planned. The phenomenon of the droplet formation on the gas side is in accordance with the observations made by Jeanty et al. [25]. These authors also reported a droplet formation on the gas side of silver-based GDEs and could verify the presence of crystalline residues of the electrolyte. On the other side, Moussallem et al. [7] observed that the droplets, which were formed on the gas side, do not always contain NaOH. They expect that, during the electrochemical process, water evaporates from the electrolyte. The water vapor diffused through the pore system of the GDE and condensates on the gas side [7]. In order to obtain assurance about the chemical composition of the droplets, more investigations are planned to investigate how different electrochemical operation parameters affect the chemical concentration of the droplets.

Figure 12 shows that, after a drop down, the saturation is increasing although no droplets form. It takes some time until new droplets form again, which is dependent on the applied potential and the GDE. This is an indication that, during the drop down of larger droplets, liquid is also sucked out of the pore system of the GDE. Subsequently, the electrolyte has to flow again in the freed areas of the GDE before new droplets can form. More detailed analyses are planned to perform at a synchrotron facility. 

Moreover, a clear relationship between the applied potential and the droplet formation is evident. Thus, the calculation of the droplet covered surface fraction (Figure 11) depicted a slower droplet formation at 0.9 V vs. RHE than at 0.2 V vs. RHE. Additionally, Figure 12b exhibits that the saturation and the new formation of the droplets after the drop down is slower for 0.9 V vs. RHE and faster for 0.2 V vs. RHE. This is in accordance with the electrochemical behavior depicted in Figure 8. Here, it is visible that higher current densities occur at higher overpotentials. Therefore, we propose that higher overpotentials lead to higher performance of the GDE due to the higher material conversion and to higher saturation behaviors than at lower overpotentials. The latter can be explained with electrowetting effects. It was found by Burchart [26] that the contact angle between the electrode surface and the electrolyte is decreasing with decreasing potential. Therefore, the wetting of the electrode is increasing [27]. This also means that the resistance against the penetration and percolation of the electrolyte is decreasing at high overpotentials. Accordingly, the electrolyte can enter and spread easier within the GDE and increases the reaction surface (three-phase boundary). It is most likely that the penetration and percolation are realized by preferred paths. However, this leads also to an increased droplet formation. Jeanty et al. [23] suggested that the occupancy of the gas side by droplets decreases the performances of the GDE because less gas enters and fewer areas for electrochemical processes are available. In the present work, a significant reduction of the electrochemical performance due to the formation of droplets could not be confirmed, although covered surface areas of about 80% were temporarily observed. However, during the chronoamperometric measurements, larger irregularities of the current densities (Figure 8) occurred at higher overpotentials, which could be an effect of the accompanying faster droplet formation on the gas side. The observation that the performance is not decreased indicates that the GDE is not flooded by the electrolyte, even though droplets form on the gas side. This indicates that, due to the high complexity of the pore system, there are some percolating paths that benefit the liquid transport to the gas side and feed the droplets. At the same time, there are enough hydrophobic paths, which are kept free of electrolyte and support the gas flow. Therefore, it is to assume that the three-phase boundary does not only form close to the surface of the electrolyte side but also can form over the entire cross section of the electrode. However, the precise distribution of the gas phase and the electrolyte is not clear. To analyze the position of the paths that are supporting the liquid and the gas transport, respectively, more detailed investigations are planned in a synchrotron facility, which offers higher resolution. 

Figure 8b shows a sudden decrease of the current density for the second cycle at 0.2 V vs. RHE. This effect is in accordance with Figure 12c, showing an unexpected inactivity within the chosen ROI at this potential. However, the reason for this behavior could not be clarified. It is to expect that a process within the pore channels leads to the sudden decrease of the performance. In this case, the spatial resolution in the laboratory X-ray is not sufficient for further analyses. 

### 4.3. Comparison of GDEs with Different Chemical Composition

**Figure 13:**
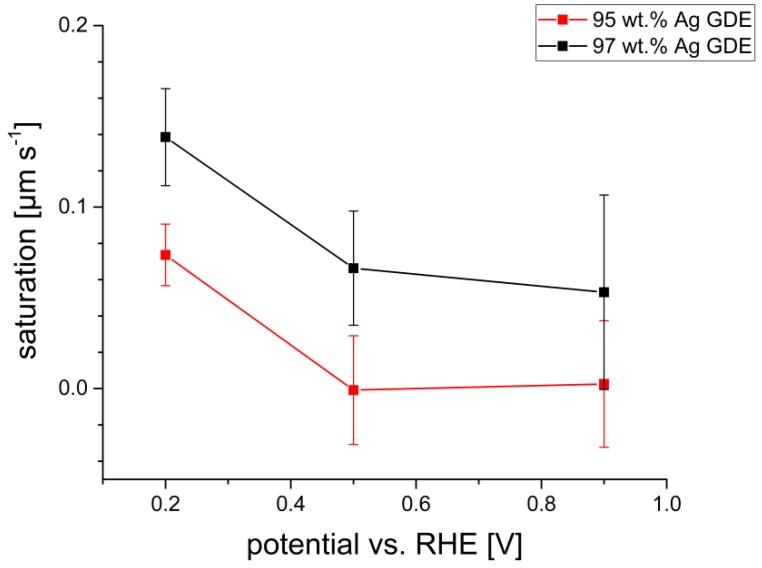
Saturation behavior of the GDE with 95 wt.% Ag (red) and the GDE with 97 wt.% Ag (black) for different applied potentials.

In general, the behavior of the different GDEs is quite similar. However, there are some differences in their electrochemical and saturation behavior. Franzen et al. [8], who compared GDEs with different chemical compositions, observed an increase of the electrochemical performance with decreasing PTFE content up to a composition of 98 wt.% Ag while, at even higher Ag contents, the performance was decreased again [8]. In this investigation, it was observed that, compared to the GDE with 95 wt.% Ag, the GDE with 97 wt.% Ag exhibits higher current densities at the same applied potentials, which indicates a higher performance and is in good agreement with the results of Franzen et al. [8]. In the present investigation, it was also observed that the saturation behavior is higher for the GDE with 97 wt.% Ag. In this context, the experimental results plotted in Figure 12 and listed in Table 1 and Table 2 show that, in general, the increase of the saturation is higher for the GDE with 97 wt.% Ag, which indicates a higher liquid transportation through the electrode into the droplets than for the GDE with 95 wt.% Ag. This can be explained by the different PTFE content of the GDEs. For the GDE with 95 wt.% Ag, the PTFE deposits at the silver grains are larger than for the GDE with 97 wt.% Ag. Therefore, the number of the pore paths that are supporting the electrolyte flow is reduced and thus the penetration and the spreading of the electrolyte within the GDE are impeded, leading to a lower saturation of the GDE. The saturation behavior of both GDEs for different potentials is summarized in Figure 13. 

## 5. Conclusions

In this investigation, operando laboratory X-ray radiography measurements were applied to analyze the effect of different potentials on the electrochemical behavior and the electrolyte distribution of two different GDEs, containing 97 wt.% Ag and 95 wt.% Ag, respectively. The applied operando experiments and the correlation of laboratory X-ray radiography and chronoamperometric measurements reveal essential processes of the GDEs. It was found that the high overpotentials lead to high saturation and faster formation of droplets due to the effect of electrowetting. Moreover, a relationship between larger pores within the Ni mesh current collector and the formation of droplets could be demonstrated. It is assumed that preferred paths form, which enables the electrolyte to penetrate the GDEs, benefit the spreading of the electrolyte within the GDEs and also support the percolation. At the same time, hydrophobic pore paths ensure the transport of the gas phase and prevent the electrolyte from completely flooding the GDE. This also suggests that the three-phase boundary can get formed in the entire cross section of the GDE. In general, the GDE with 97 wt.% Ag shows better saturation behavior than the GDE with 95 wt.% Ag.

## Figures and Tables

**Table 1:** Saturation behavior and visible effects for different potentials and cycles for GDE with 95 wt.% Ag.

Potential [V]	Cycle	Saturation [µm·s^−1^]	Imaging
0.9 vs. RHE	first	decrease	no changes of the electrolyte distribution
0.5 vs. RHE			
0.2 vs. RHE		increase of 0.094	small droplets form
0.9 vs. RHE	second	almost constant	no changes of droplets
0.5 vs. RHE			
0.2 vs. RHE		increase of 0.063	small droplets agglomerate
0.9 vs. RHE	third	increase of 0.033	no changes of droplets
0.5 vs. RHE		sudden decrease	drop down of large droplets
0.2 vs. RHE		increase of 0.080	new droplets form
0.9 vs. RHE	fourth	sharp peak	peak caused by a large droplet dropping into the ROI; apart from this saturation constant
0.5 vs. RHE		increase of 0.027	droplets agglomerate; in the end of this cycle, droplets dropped down
0.2 vs. RHE		increase of 0.062	no changes

**Table 2:** Saturation behavior and visible effects for different potentials and cycles for GDE with 97 wt.% Ag, ROI 1.

Potential [V]	Cycle	Saturation [µm·s^−1^]	Imaging
0.9 vs. RHE	first	decrease	no changes of the electrolyte distribution
0.5 vs. RHE			
0.2 vs. RHE		sudden increase	small droplets form
0.9 vs. RHE	second	increase of 0.094	no changes of droplets
0.5 vs. RHE		increase of 0.099	no changes of droplets
0.2 vs. RHE		increase of 0.145	droplets agglomerate
0.9 vs. RHE	third	almost constant	droplets coagulate and drop down, afterwards no changes of the electrolyte distribution
0.5 vs. RHE		increase of 0.022	no changes of the electrolyte distribution
0.2 vs. RHE		increase of 0.107	new droplets form
0.9 vs. RHE	fourth	saturation increases	caused by a droplet, which dropped into ROI
0.5 vs. RHE		increase of 0.056	drop down in the end of this cycle
0.2 vs. RHE		increase of 0.121	no changes of the electrolyte distribution

## References

[1] Kintrup J., Millaruelo M., Trieu V., Bulan A., Mojica E.S. (2017). Gas Diffusion Electrodes for Efficient Manufacturing of Chlorine and Other Chemicals. Electrochem. Soc. Interface.

[2] Moussallem I., Jörissen J., Kunz U., Pinnow S., Turek T. (2008). Chlor-alkali electrolysis with oxygen depolarized cathodes: History, present status and future prospects. J. Appl. Electrochem..

[3] Jörissen J., Turek T., Weber R. (2011). Chlorherstellung mit Sauerstoffverzehrkathoden. Energieeinsparung bei der Elektrolyse. Chem. Unserer Zeit.

[4] Garcia-Herrero I., Margallo M., Onandía R., Aldaco R., Irabien A. (2017). Environmental challenges of the chlor-alkali production: Seeking answers from a life cycle approach. Sci. Total Environ..

[5] Jung J., Postels S., Bardow A. (2014). Cleaner chlorine production using oxygen depolarized cathodes? A life cycle assessment. J. Clean. Prod..

[6] Chatenet M., Genies-Bultel L., Aurousseau M., Durand R., Andolfatto F. (2002). Oxygen reduction on silver catalysts in solutions containing various concentrations of sodium hydroxide—Comparison with platinum. J. Appl. Electrochem..

[7] Moussallem I., Pinnow S., Wagner N., Turek T. (2012). Development of high-performance silver-based gas-diffusion electrodes for chlor-alkali electrolysis with oxygen depolarized cathodes. Chem. Eng. Process. Intensif..

[8] Franzen D., Ellendorff B., Paulisch M.C., Hilger A., Osenberg M., Manke I., Turek T. (2019). Influence of binder content in silver-based gas diffusion electrodes on pore system and electrochemical performance. J. Appl. Electrochem..

[9] Al Rwashdeh S.S., Manke I., Markötter H., Klages M., Göbel M., Haußmann J., Scholta J., Banhart J. (2017). In Operando Quantification of Three-Dimensional Water Distribution in Nanoporous Carbon-Based Layers in Polymer Electrolyte Membrane Fuel Cells. ACS Nano.

[10] Boillat P., Frei G., Lehmann E.H., Scherer G.G., Wokaun A. (2010). Neutron Imaging Resolution Improvements Optimized for Fuel Cell Applications. Electrochem. Solid State Lett..

[11] Fazeli M., Hinebaugh J., Fishman Z., Tötzke C., Lehnert W., Manke I., Bazylak A. (2016). Pore network modeling to explore the effects of compression on multiphase transport in polymer electrolyte membrane fuel cell gas diffusion layers. J. Power Sources.

[12] Manke I., Hartnig C., Grünerbel M., Lehnert W., Kardjilov N., Haibel A., Hilger A., Banhart J., Riesemeier H. (2007). Investigation of water evolution and transport in fuel cells with high resolution synchrotron X-ray radiography. Appl. Phys. Lett..

[13] Markötter H., Manke I., Krüger P., Arlt T., Haussmann J., Klages M., Riesemeier H., Hartnig C., Scholta J., Banhart J. (2011). Investigation of 3D water transport paths in gas diffusion layers by combined in-situ synchrotron X-ray radiography and tomography. Electrochem. Commun..

[14] Sasabe T., Deevanhxay P., Tsushima S., Hirai S. (2011). Investigation on the effect of microstructure of proton exchange membrane fuel cell porous layers on liquid water behavior by soft X-ray radiography. J. Power Sources.

[15] Schröder A., Wippermann K., Lehnert W., Stolten D., Sanders T., Baumhöfer T., Kardjilov N., Hilger A., Banhart J., Manke I. (2010). The influence of gas diffusion layer wettability on direct methanol fuel cell performance: A combined local current distribution and high resolution neutron radiography study. J. Power Sources.

[16] Gebhard M., Paulisch M., Hilger A., Franzen D., Ellendorff B., Turek T., Manke I., Roth C. (2019). Design of an In-Operando Cell for X-ray and Neutron Imaging of Oxygen-Depolarized Cathodes in Chlor-Alkali Electrolysis. Materials.

[17] Moussallem I. (2011). Development of Gas Diffusion Electrodes for a New Energy Saving Chlor-Alkali Electrolysis Process. Ph.D. Thesis.

[18] Kardjilov N., Hilger A., Manke I. (2016). CONRAD-2: Cold Neutron Tomography and Radiography at BER II (V7). J. Large Scale Res. Facil..

[19] Kardjilov N., Hilger A., Manke I., Woracek R., Banhart J. (2016). CONRAD-2: The new neutron imaging instrument at the Helmholtz-Zentrum Berlin. J. Appl. Crystallogr..

[20] Schindelin J., Arganda-Carreras I., Frise E., Kaynig V., Longair M., Pietzsch T., Preibisch S., Rueden C., Saalfeld S., Schmid B. (2012). Fiji: An open-source platform for biological-image analysis. Nat. Methods.

[21] Legland D., Arganda-Carreras I., Andrey P. (2016). MorphoLibJ: Integrated library and plugins for mathematical morphology with ImageJ. Bioinformatics.

[22] Kunz P., Paulisch M., Osenberg M., Bischof B., Manke I., Nieken U. (2019). Prediction of Electrolyte Distribution in Technical Gas Diffusion Electrodes: From Imaging to SPH Simulations. Transp. Porous Med..

[23] Haußmann J., Markötter H., Alink R., Bauder A., Dittmann K., Manke I., Scholta J. (2013). Synchrotron radiography and tomography of water transport in perforated gas diffusion media. J. Power Sources.

[24] Alink R., Haußmann J., Markötter M., Schwager M., Manke I., Gerteisen D. (2013). The Influence of Porous Transport Layer Modifications on the Water Management in PEM Fuel Cells. J. Power Sources.

[25] Jeanty P., Scherer C., Magori E., Wiesner-Fleischer K., Hinrichsen O., Fleischer M. (2018). Upscaling and continuous operation of electrochemical CO_2_ to CO conversion in aqueous solutions on silver gas diffusion electrodes. J. CO_2_ Util..

[26] Burchardt T. (2004). An evaluation of electrocatalytic activity and stability for air electrodes. J. Power Sources.

[27] Bidault F., Brett D., Middleton P.H., Brandon N.P. (2009). Review of gas diffusion cathodes for alkaline fuel cells. J. Power Sources.

